# Necroptosis plays a role in TL1A-induced airway inflammation and barrier damage in asthma

**DOI:** 10.1186/s12931-024-02900-4

**Published:** 2024-07-10

**Authors:** Xiaofei Liu, Jintao Zhang, Dong Zhang, Yun Pan, Rong Zeng, Changjuan Xu, Shuochuan Shi, Jiawei Xu, Qian Qi, Xueli Dong, Junfei Wang, Tian Liu, Liang Dong

**Affiliations:** 1 Department of Respiratory, Shandong Institute of Respiratory Diseases, The First Affiliated Hospital of Shandong First Medical University, Jinan, 250014 China; 2 Department of Respiratory, Shandong Provincial Qianfoshan Hospital, Shandong University, Jinan, China; 3 Department of Respiratory and Critical Care Medicine, Qilu Hospital of Shandong University, Jinan, China

**Keywords:** TL1A, Necroptosis, MLKL, Airway inflammation, Tight junction, NF-κB

## Abstract

**Background:**

Airway epithelial cell (AEC) necroptosis contributes to airway allergic inflammation and asthma exacerbation. Targeting the tumor necrosis factor-like ligand 1 A (TL1A)/death receptor 3 (DR3) axis has a therapeutic effect on asthmatic airway inflammation. The role of TL1A in mediating necroptosis of AECs challenged with ovalbumin (OVA) and its contribution to airway inflammation remains unclear.

**Methods:**

We evaluated the expression of the receptor-interacting serine/threonine-protein kinase 3(RIPK3) and the mixed lineage kinase domain-like protein (MLKL) in human serum and lung, and histologically verified the level of MLKL phosphorylation in lung tissue from asthmatics and OVA-induced mice. Next, using MLKL knockout mice and the RIPK3 inhibitor GSK872, we investigated the effects of TL1A on airway inflammation and airway barrier function through the activation of necroptosis in experimental asthma.

**Results:**

High expression of necroptosis marker proteins was observed in the serum of asthmatics, and necroptosis was activated in the airway epithelium of both asthmatics and OVA-induced mice. Blocking necroptosis through MLKL knockout or RIPK3 inhibition effectively attenuated parabronchial inflammation, mucus hypersecretion, and airway collagen fiber accumulation, while also suppressing type 2 inflammatory factors secretion. In addition, TL1A/ DR3 was shown to act as a death trigger for necroptosis in the absence of caspases by silencing or overexpressing TL1A in HBE cells. Furthermore, the recombinant TL1A protein was found to induce necroptosis in vivo, and knockout of MLKL partially reversed the pathological changes induced by TL1A. The necroptosis induced by TL1A disrupted the airway barrier function by decreasing the expression of tight junction proteins zonula occludens-1 (ZO-1) and occludin, possibly through the activation of the NF-κB signaling pathway.

**Conclusions:**

TL1A-induced airway epithelial necroptosis plays a significant role in promoting airway inflammation and barrier dysfunction in asthma. Inhibition of the TL1A-induced necroptosis pathway could be a promising therapeutic strategy.

**Supplementary Information:**

The online version contains supplementary material available at 10.1186/s12931-024-02900-4.

## Introduction

Asthma is a chronic airway inflammatory disease that causes respiratory symptoms including wheezing, dyspnea, coughing, and chest tightness, as well as airflow restrictions. These symptoms result from underlying mechanisms such as airway inflammation, airway hyperresponsiveness, mucus hypersecretion, and airway remodeling [1]. Asthma affects over 300 million people globally and has become a significant public health issue with a considerable healthcare burden that needs to be seriously addressed [2]. Although asthma mortality among adults and children has declined globally due to the increased use of inhaled corticosteroids over recent decades, the management of severe asthma that does not respond to corticosteroids remains a significant challenge. Additionally, the resolution of asthma-related hospitalizations is proving to be challenging. Therefore, a more comprehensive understanding of the pathophysiology of asthma is needed to develop new therapeutic options for people with asthma.

Necroptosis is a recently characterized form of programmed cell death that entails activating the RIPK3 protein and subsequently phosphorylating MLKL by RIPK3, resulting in a conformational shift in MLKL [3]. Programmed cell necrosis has been shown to induce inflammatory pathologies in various tissues, and the regulation of epithelial cell death has emerged as a critical mechanism modulating barrier homeostasis of airway, skin, and intestinal epithelial surfaces [4–6]. AEC is a primary target for numerous inhalants and inflammatory mediators. AEC death contributes to allergic airway inflammation and immune response and causes damage to epithelial structures [4]. However, necrosis-induced airway epithelial changes in asthma and the underlying mechanisms have not been fully elucidated.

The function of TL1A in association with asthma has been the subject of recent reports. TL1A, in combination with its cognate receptor DR3, is a crucial contributor to the initiation of allergic lung inflammation. It is reported that constitutive TL1A prompts DR3-rich group 2 innate lymphoid cells (ILC2) to generate Th2 cytokines, including IL-13 [7, 8]. Secretory TL1A has been reported to be increased in the sputum of asthmatics and the bronchoalveolar lavage fluid (BALF) of OVA-induced mice [9, 10]. Previous studies, including our own, have detailed a strong correlation between highly expressed secretory and constitutive TL1A/DR3 with airway remodeling and inflammation in asthma [9, 11, 12].

Interestingly, Bittner S et al. identified DR3 as a new death receptor capable of triggering necroptosis in addition to tumor necrosis factor receptor-1(TNFR1), FAS, pattern recognition receptors (PRRs), and toll-like receptor 3(TLR3) [13]. However, the role of TL1A in inducing necroptosis in the pathogenesis of asthma has not been investigated. For the first time, we described the role of TL1A and necroptosis signaling in the pathogenesis of asthma using lung tissues and serum samples from healthy controls and asthmatics, a programmed necroptosis cell model of airway epithelium, and an OVA-induced mouse model of asthma.

## Methods

### Human samples

Serum samples were obtained from patients diagnosed with asthma according to the Global Initiative for Asthma (GINA) in the respiratory department of the First Affiliated Hospital of Shandong First Medical University. Healthy control serums were obtained from the physical examination department. Asthmatic lung tissues were obtained from the bronchoscopy biopsy, and healthy control lung tissues were obtained from patients with benign pulmonary nodules with normal lung function and no other respiratory diseases. This work has been approved by the Ethics Committee of Shandong First Medical University (ethics review number: 2021-S923). All participants entering the study provided written informed consent.

### Cell culture and transfection

The human bronchial epithelial cell line (HBE) was derived from Fuheng Biology (Shanghai, China) and cultured in KM medium containing 1% KGS. HBE cells were transfected with overexpression control plasmid vector (OE-control) or overexpression membrane-bound TL1A plasmid vector (OE-TL1A), small interfering RNA negative control(siNC) or small interfering RNA targeting TL1A(siTL1A) using the EndoFectin™ Max Transfection Reagent (Genecopoeia, China) according to the manufacturer’s instructions. The siRNA and the plasmids sequence were described in the previous article [9]. Cells were pretreated with 5 µM BAY 11-7082 for 1 h to block NF-κB before the addition of TSZ (Beyotime, China).

### Animals

Six- to eight-week-old male and female MLKL knockout (house on the C57BL/6 N background) mice were derived by Cyagen Biosciences. Wild-type (WT) C57BL/6 N mice matched in age and weight were obtained from Beijing Vital River Laboratory Animal Technology. The methods of asthma model construction, RIPK3 inhibitor GSK872 (Selleck, China) injection, and recombinant TL1A (R&D systems, USA) nasal administration were illustrated in the figures below.

### Bronchoalveolar lavage cell counts and protein concentration

The BALF procedure involved rinsing the lungs 3 times with 1 mL of PBS through a tracheal cannula. Cell-free supernatant was stored at -20℃ before analysis of Enzyme-Linked Immunosorbent Assay (ELISA) and Bicinchoninic Acid Assay (BCA). Red blood cell (RBC) lysis buffer was used to remove residual RBCs and the remaining cells were counted by trypan blue staining.

### ELISA assay

RIPK3 and p-MLKL protein levels were measured in human serum using a commercially available ELISA kit purchased from CUSABIO and RayBiotech, as described by the manufacturer. Meanwhile, protein levels of IL-4, IL-5, and IL-13 in mouse serum and BALF were detected using ELISA kits (MultiSciences, China) according to the manufacturer’s instructions. All samples were measured in duplicate.

### Lung histology

The human lung biopsy tissues and mouse lung tissues were fixed in 4% paraformaldehyde for 48 h at room temperature (RT) and paraffin-embedded. After sectioning in 4 μm, the tissues were deparaffinized for use in hematoxylin and eosin (H&E), periodic acid-schiff (PAS), and Masson trichrome staining. Lung inflammation and PAS scoring were performed in a blinded fashion. Standard scoring was performed according to the method described by Qin et al [14]. and Padrid et al [15].

### CCK8 assay

HBE cells were seeded in 96-well-plates, treated with BV6 (0.5 µM, 2 h), and zVAD-fmk (20 µM, 2 h), and subsequently challenged with the specified concentration of recombinant TL1A protein (0, 10, 100, 500, 1000 ng/mL) for 18 h. The treated cells were added with 10 µL CCK8 reagent per well and incubated for 2 h. The absorbance at 450 nm was measured using a Microplate Reader (Thermo Fisher Scientific, USA). The wells without added cells were used as blank control and three replicates were set up in each well.

### RNA isolation and real-time PCR(RT-PCR)

Total RNA was isolated from HBE cells using the RNA Fast 200 Extraction Kit (Fastagen Biotech, China) according to the manufacturer’s protocol. One microgram of total RNA was used to generate cDNA using HiScript III SuperMix for qPCR (+ gDNA wiper) (Vazyme, China). Real-time PCR was performed for IL-1β, IL-6, IL-8, monocyte chemotactic protein-1(MCP-1), MLKL, RIPK3, vimentin, α-SMA, TL1A, and GAPDH using ChamQ Universal SYBR qPCR Master Mix (Vazyme, China). The primers used are described in supplementary Table 1.

### Western blot analysis

Total protein was extracted from cultured HBE cells and lung tissues by using RIPA lysis buffer containing 1% PMSF and 1% protein phosphatase inhibitor. Protein concentration was measured using a BCA Protein Assay kit (Beyotime, China). Equal amounts of protein were separated on 10% SDS- polyacrylamide gels and transferred to PVDF membranes. The desired target bands were blocked in 5% skimmed milk for 1 h at RT and subsequently probed with the desired primary antibody overnight at 4 °C. The following antibodies were used: anti-RIPK1 (ET1701-79, HUABIO), anti-Phospho-RIPK1 (65,746, CST), anti-RIPK3 (ET1901-27, HUABIO), anti-Phospho-RIPK3 (ab195117, ab209384; Abcam), anti-MLKL (ab184718, Abcam), anti-Phospho-MLKL (ab187091, Abcam; 37,333, Cell Signaling Technology), anti-NF-κB p65 (8242, CST), anti-Phospho-NF-κB p65 (AF2006, Affinity), anti-IκBα (9242, CST), anti-Phospho-IκBα (2859, CST), anti-ZO-1 (ab216880, Abcam), anti-occludin (ab216327, Abcam), anti-GAPDH (ab9485, Abcam), anti-TL1A (ab85566; Abcam), anti-fibronectin (ET1702-25, HUABIO), anti-alpha smooth muscle actin (ET1607-53, HUABIO), anti-N-cadherin (13,116, CST). The dilution ratio of the antibodies used for western blotting is 1:1 000. After 3 washes in TBST, the bands were incubated for 1 h with HRP-conjugated secondary antibodies (HA1001, HUABIO, 1:5 000). The bands were washed 3 times again in TBST and analyzed using a ChemiDoc gel imaging system (Bio-Rad, USA).

### Immunofluorescence

The HBE cells were propagated in 24-well culture plates using cell-climbing slices. Following the indicated treatments, cells were washed with PBS, fixed with 4% paraformaldehyde, and then incubated in 0.5% Triton solution. Subsequently, cells were blocked with 1% bovine serum albumin for 1 h at RT before being incubated with rabbit anti-Phospho-MLKL primary antibodies (1:200) at 4 °C overnight. After washed 3 times with PBS, the cells were incubated with FITC goat anti-rabbit IgG (Abbkine, China, 1:400) at RT for 1 h. The cell nuclei were stained with DAPI (Sigma, USA, 1:400) for 1 min. Images are visualized with a Leica upright fluorescence microscope (Leica, Germany).

### Immunohistochemistry

Paraffin-embedded sections were deparaffinized and incubated in citrate buffer at pH 6.0 for 15 min at 95 °C. The sections were then washed in water for 5 min and incubated in 3% H_2_O_2_ for 10 min to inhibit peroxidase. Sections were blocked in 5% BSA for 30 min at RT in a wet box and incubated with primary antibody overnight at 4 °C. The dilution ratio of the antibodies used is as follows: anti-Cleaved Caspase-3 (AF7022, Affinity, 1:1000), anti-ZO-1 (ab216880, Abcam, 1:1000), and anti-occludin (ab216327, Abcam, 1:200). Sections were washed 3 times with PBS and then incubated with rabbit secondary antibody for 30 min at RT. Antigens were detected using a DAB detection kit as recommended by the supplier. Finally, the sections were stained with hematoxylin for visualization.

### Immunofluorescent TUNEL staining

The TUNEL assay kit (Meilunbio, China) was used. After deparaffinization, 20 µg/mL Proteinase K was added to the sections for 20 min at 37 °C, and washed 3 times with PBS for 5 min each time. TUNEL solution (TdT enzyme: FITC-12-dUTP labeling mix = 1:9) was added dropwise to the samples and incubated for 1 h at 37 °C. The sections were washed 3 times with PBS. The nuclei were then stained with DAPI (Sigma, USA). Finally, the sections were sealed with an anti-fluorescence attenuation sealer.

### RNA-sequencing analysis

Fresh lung tissues of OVA-challenged WT and MLKL knockout mice were collected and immediately stored at -80 °C for RNA extraction. RNA sequencing was performed by Huada Gene (Shenzhen, China).

### Statistical analysis

Statistical analysis was conducted using GraphPad Prism 8 and IBM SPSS Statistics 22 software. Normality was assessed using the Shapiro-Wilk test. For human studies, data were presented as median (IQR) and analyzed by the Mann-Whitney U test. For mouse studies, data were presented as mean ± SEM and analyzed by unpaired t-test or one-way ANOVA with correction by Tukey’s test. *P* < 0.05 was considered a statistically significant difference. All experiments were designed with more than 3 replicates.

## Results

### Enhanced necroptosis is observed in the airway epithelium of both asthmatics and OVA-induced mice

**Fig. 1:**
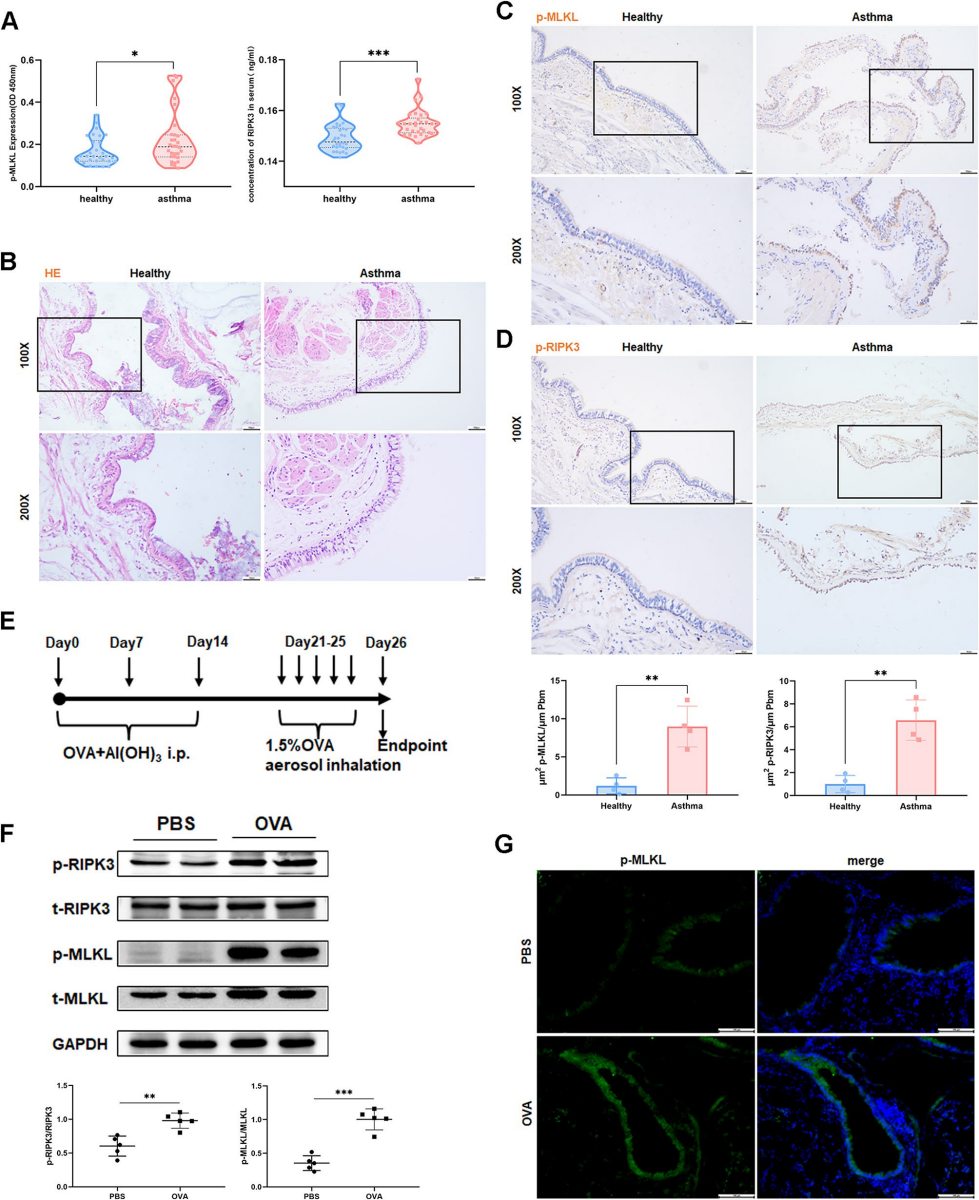
Necroptosis is enhanced in the airway epithelium of asthmatics and OVA-induced mice. **(A)** p-MLKL and RIPK3 concentrations in the plasma of healthy control subjects (*n* = 30) and asthmatics (*n* = 30) were measured by ELISA. **(B)** Representative images of H&E staining in human lung tissue sections (*n* = 4). **(C, D)** Representative images of p-MLKL and p-RIPK3 immunohistochemical staining in human lung tissue sections (*n* = 4). **(E)** Experimental scheme for OVA-induced mice model of asthma. **(F)** Western blot analysis of p-RIPK3, t-RIPK3, p-MLKL, and t-MLKL in lungs of OVA-induced mice (*n* = 5). **(G)** Lung slides from OVA-induced mice were immunofluorescence stained with anti-p-MLKL (*n* = 4). Bars = 100 μm, Bars = 50 μm.*p < 0.05, **p < 0.01, ***p < 0.001

To explore the role of necroptosis in asthma, serum samples from 30 healthy control subjects and 30 asthmatics were collected (Supplementary Table 2). Serum concentrations of p-MLKL and RIPK3 were significantly elevated in asthma patients compared to healthy controls (Fig. 1A), but no significant change in the level of HMGB1 was observed (data not shown). We compared p-MLKL and p-RIPK3 protein levels in human lung tissue sections obtained from 4 healthy controls and 4 asthma patients (Fig. 1B). Quantitative analysis revealed significantly higher levels of p-MLKL and p-RIPK3 positive staining in the airway epithelium of asthmatic patients than in healthy controls (Fig. 1C, D). To extend these findings, we investigated the manifestation of necroptosis marker proteins in the lungs of the OVA-challenged mice (Fig. 1E and F). Additionally, lung tissue immunofluorescence analysis illustrated a heightened expression of p-MLKL in the airway epithelium, consistent with the results observed in human samples (Fig. 1G).

To determine increased necrosis rather than apoptosis of airway epithelial cells after OVA stimulation, we performed TUNEL and cleaved caspase 3 (CC3) staining. The number of TUNEL + cells was increased in the lungs of OVA-exposed mice, both in the airway epithelium and in peripheral inflammatory cells. However, there was no difference in CC3 + cells in the airways, suggesting that necroptosis occurred in the epithelium (Supplementary Fig. 1A).

### Pharmacological blockage and genomic deletion of necrotic signaling ameliorate airway inflammation and remodeling in asthma

**Fig. 2:**
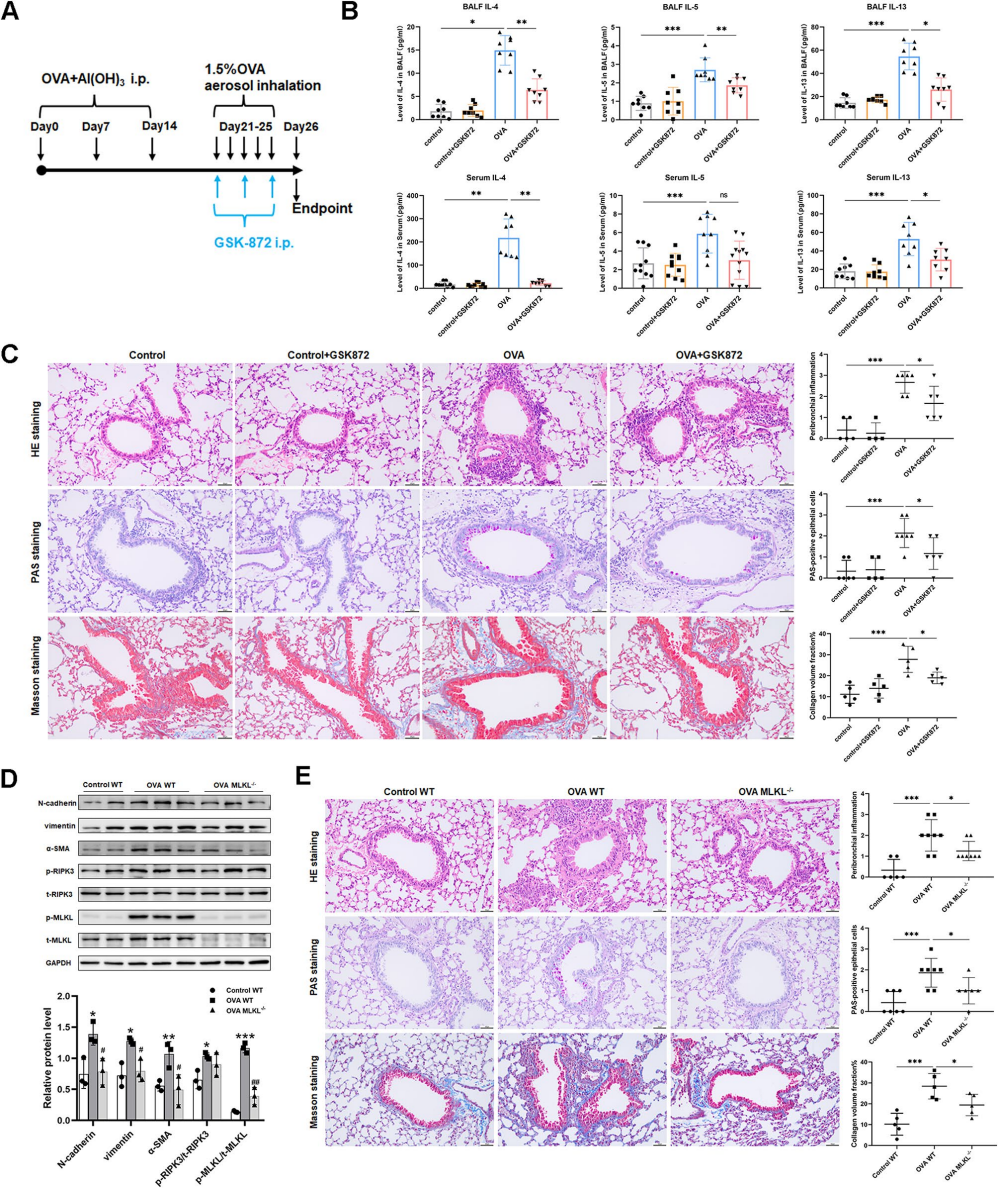
Blocking necrotic signaling can alleviate airway inflammation and remodeling in asthma. **(A)** OVA-induced asthma mouse model experimental scheme and GSK872 dosing schedule. **(B)** IL-4, IL-5, and IL-13 concentrations in BALF and serum of mice measured by ELISA (*n* = 8–10). **(C)** Representative photomicrographs for lung paraffin sections from mice stained with H&E, PAS, and Masson staining, along with quantification of inflammation scores, PAS-positive cells, and collagen deposition around the airways (*n* = 5–8). **(D)** Western blot analysis of N-cadherin, vimentin, α-SMA, p-RIPK3, t-RIPK3, p-MLKL, and t-MLKL in lungs of WT and MLKL^−/−^ mice (*n* = 3). *control WT vs.*. *OVA WT*: *p < 0.05, **p < 0.01, ***p < 0.001. *OVA WT vs.*. *OVA MLKL*^*−/−*^: #*p* < 0.05, ##*p* < 0.01, ###*p* < 0.001. **(E)** Representative photomicrographs for lung paraffin sections from mice stained with H&E, PAS, and Masson staining, along with quantification of inflammation scores, PAS-positive cells, and collagen deposition around the airways (*n* = 5–8). Bars = 50 μm.*p < 0.05, **p < 0.01, ***p < 0.001, and ns. non-significant

To clarify the critical function of necroptosis in OVA-induced airway inflammation and remodeling, we performed experiments to block necroptosis signaling in vivo. By administering the RIPK3 inhibitor GSK872 via intraperitoneal injection on days 21, 23, and 25, 1 h before the OVA challenge, a significant reduction in the release of type 2 inflammatory factors IL-4, IL-5, and IL-13 in BALF and serum was observed (Fig. 2A, B). In addition, lung histopathologic analysis confirmed that GSK872-treated mice exhibited a notable reduction in OVA-induced airway inflammation, mucus hypersecretion, and collagen deposition (Fig. 2C). Genetic disruption of the necroptosis signaling pathway reduced airway remodeling markers N-cadherin, vimentin, and α-SMA at the protein level and alleviated airway inflammation, mucus hypersecretion, and collagen deposition in OVA-challenged mice (Fig. 2D, E). In conclusion, the OVA challenge activates the MLKL-dependent necroptosis pathway. Inhibition of necroptosis by GSK872 or MLKL knockout ameliorated OVA-induced asthma changes.

### TL1A is essential for the activation of necroptosis in HBE cells

**Fig. 3:**
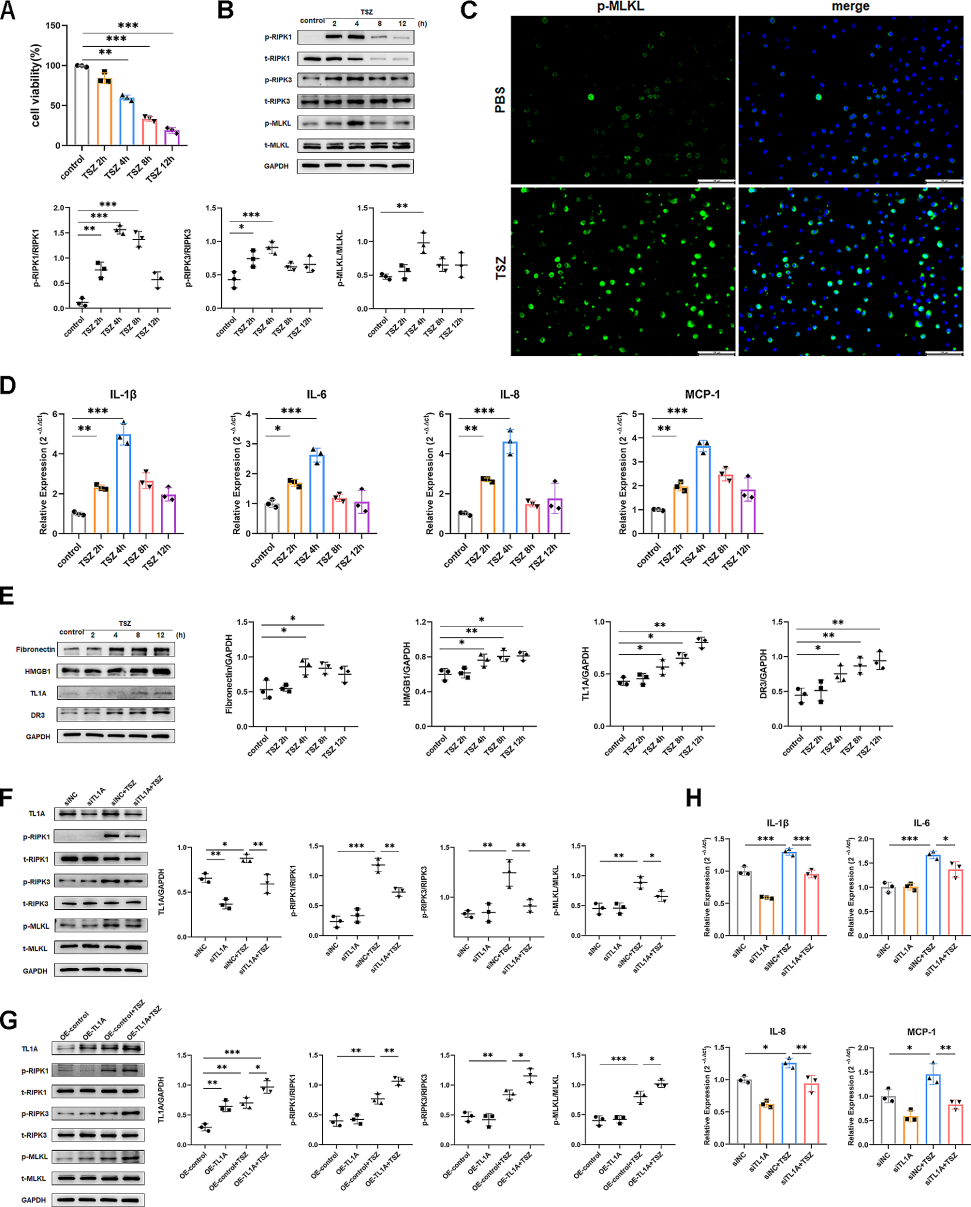
TL1A is critical for HBE cell necroptosis and the production of inflammatory factors. **(A, B, D, E)** HBE cells were treated with TSZ for 2,4,8,12 h. **(A)** Viability was determined by CCK8. **(B)** p-RIPK1, t-RIPK1, p-RIPK3, t-RIPK3, p-MLKL, and t-MLKL expression were determined by Western blot analysis in total protein of cell lysates. **(C)** HBE cells were treated with TSZ for 4 h and stained by immunofluorescence for p-MLKL. **(D)** mRNA expression of IL-1β, IL-6, IL-8, and MCP-1 was measured by RT-PCR relative to the expression of reference genes GAPDH. **(E)** fibronectin, HMGB1, TL1A, and DR3 expression were determined by Western blot analysis in total protein of cell lysates. **(F)** HBE cells were transfected with siTL1A and then stimulated by TSZ for 4 h. P-RIPK1, t-RIPK1, p-RIPK3, t-RIPK3, p-MLKL, and t-MLKL expression were determined by Western blot. **(G)** HBE cells were transfected with OE-TL1A and then stimulated by TSZ for 4 h. P-RIPK1, t-RIPK1, p-RIPK3, t-RIPK3, p-MLKL, and t-MLKL expression were determined by Western blot. **(H)** mRNA expression of IL-1β, IL-6, IL-8, and MCP-1 were measured by RT-PCR after siTL1A transfection. *N* = 3. Bars = 100 μm.*p < 0.05, **p < 0.01, ***p < 0.001

To investigate the mechanism of necroptosis and TL1A in asthma, we established an in vitro cell model of airway epithelial necroptosis using TSZ (TNF-α, Smac mimetic, zVAD-fmk). This combination activates the cell death pathway through TNF-α, which generates death signals, but inhibits caspases, antagonizes cIAP and XIAP activation, and therefore triggers necroptosis instead of apoptosis [16, 17]. The viability of TSZ-treated HBE cells decreased over time, objectively indicating reduced cell health (Fig. 3A). Phosphorylation of RIPK1, RIPK3, and MLKL in HBE cells was induced by TSZ and increased over time, peaking at 4 h of treatment (Fig. 3B, C). However, phosphorylation levels decreased at longer treatment time due to the reduced ability of cells to synthesize proteins late in necroptosis. The mRNA levels of the inflammatory factors IL-1β, IL-6, IL-8, and MCP-1 corresponded with the changes observed in the necroptosis marker proteins (Fig. 3D). Besides, an increase in TL1A and DR3 protein expression was observed, consistent with the increase in fibronectin and HMGB1 (Fig. 3E).

To examine the role of TL1A in necroptosis and inflammatory process in bronchial epithelial cells, we transfected HBE cells with siTL1A. The results revealed that reduced TL1A protein expression did not affect p-RIPK1, p-RIPK3, and p-MLKL expression without TSZ treatment. After TSZ treatment, TL1A downregulated cells showed decreased levels of p-RIPK1, p-RIPK3 and p-MLKL compared to siNC transfected cells (Supplementary Fig. 2A, Fig. 3F). Meanwhile, TL1A knockdown significantly decreased the expression of IL-1β, IL-6, IL-8, and MCP-1, which are promoted by the activation of necroptosis induced by TSZ in HBE cells (Fig. 3H). In addition, we constructed a TL1A over-expression plasmid (OE-TL1A) to explore the function of membrane-bound TL1A in necroptosis (Supplementary Fig. 2B). Our observations revealed that elevated expression of membrane-bound TL1A enhanced TSZ-induced phosphorylation of RIPK1, RIPK3, and MLKL (Fig. 3G). Taken together, these results indicate that both secretory and constitutive TL1A are associated with airway epithelial necroptosis.

### Administration of TL1A directly induces features of necroptosis

**Fig. 4:**
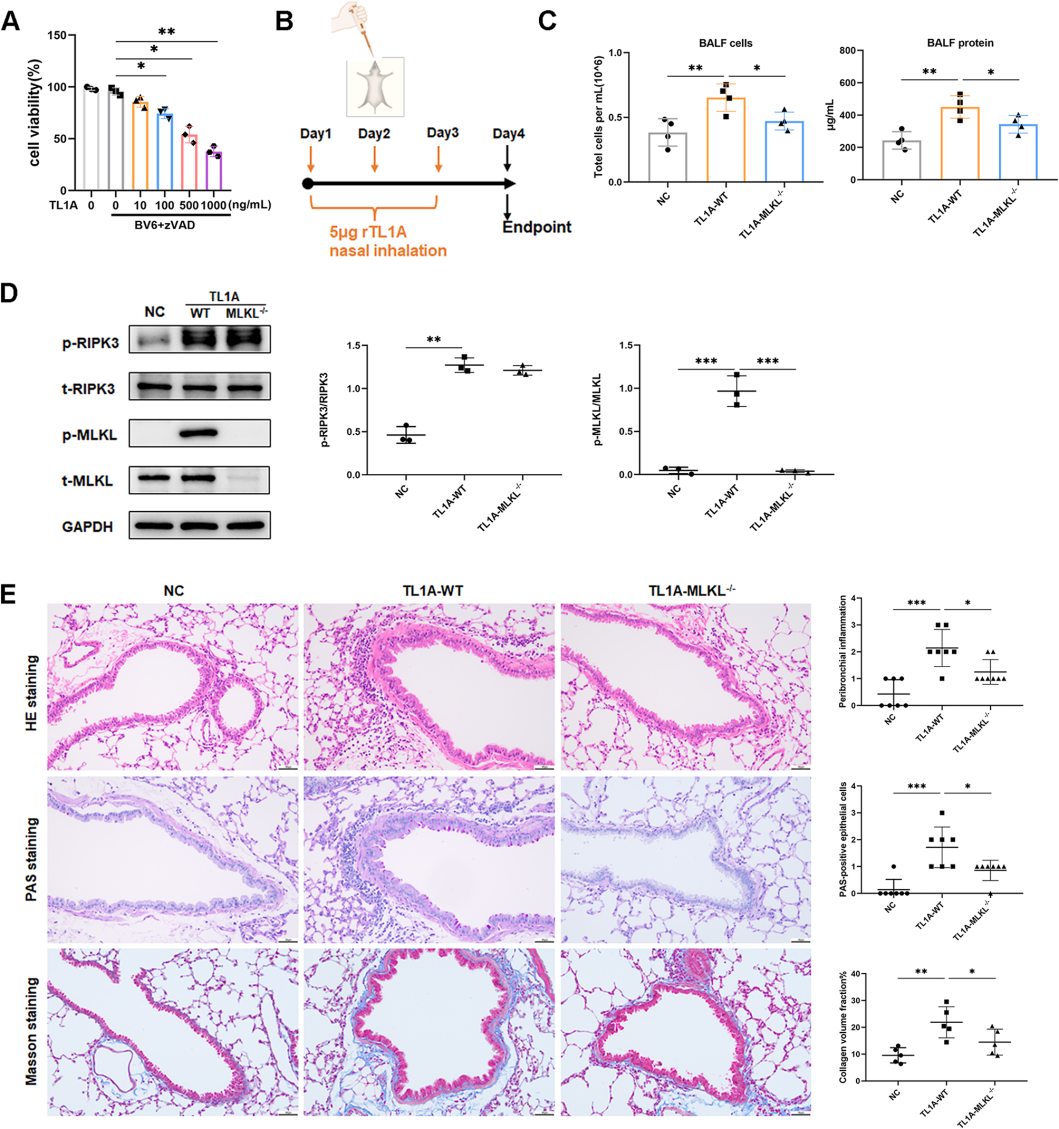
Administration of TL1A directly induces features of necroptosis. **(A)** HBE cells were treated with BV6 (0.5 µM) and zVAD-fmk (20 µM) for 2 h, followed by stimulation with TL1A for 18 h. Viability was determined by CCK8 (*n* = 3). **(B)** The dosing regimen of recombinant protein TL1A. **(C)** Total number of cells and total protein content in BALF (*n* = 4). **(D)** Western blot analysis of p-RIPK3, t-RIPK3, p-MLKL, and t-MLKL in lungs of mice (*n* = 3). **(E)** Representative photomicrographs for lung paraffin sections from mice stained with H&E, PAS, and Masson staining, along with quantification of inflammation scores, PAS-positive cells, and collagen deposition around the airways (*n* = 5–8). Bars = 50 μm.*p < 0.05, **p < 0.01, ***p < 0.001

Necroptosis induced by TL1A occurred in a dose-dependent manner in the presence of BV6 (cIAP and XIAP inhibitor) and zVAD-fmk (caspase inhibitor) in vitro (Fig. 4A). To observe the induction of necroptosis by TL1A in vivo, we administered 5 µg of recombinant TL1A protein per day to mice via nasal drips for 3 days (Fig. 4B). TL1A intervention triggered a programmed necrotic process in the lungs of mice, leading to an elevation in total cell count and protein content in BALF. However, the deletion of the MLKL gene significantly alleviated these pathological changes in the lungs (Fig. 4C and D). Intranasal administration of TL1A induced parabronchial inflammation, mucus hypersecretion, and fibrosis features in the lung, which can be reversed by MLKL knockout (Fig. 4E). After TL1A treatment, there was a significant increase in TUNEL + cells, while no difference was observed in CC3 + cells, suggesting that epithelial cells died by necroptosis (Supplementary Fig. 1C).

### TL1A-induced necroptosis promotes asthma inflammation

**Fig. 5:**
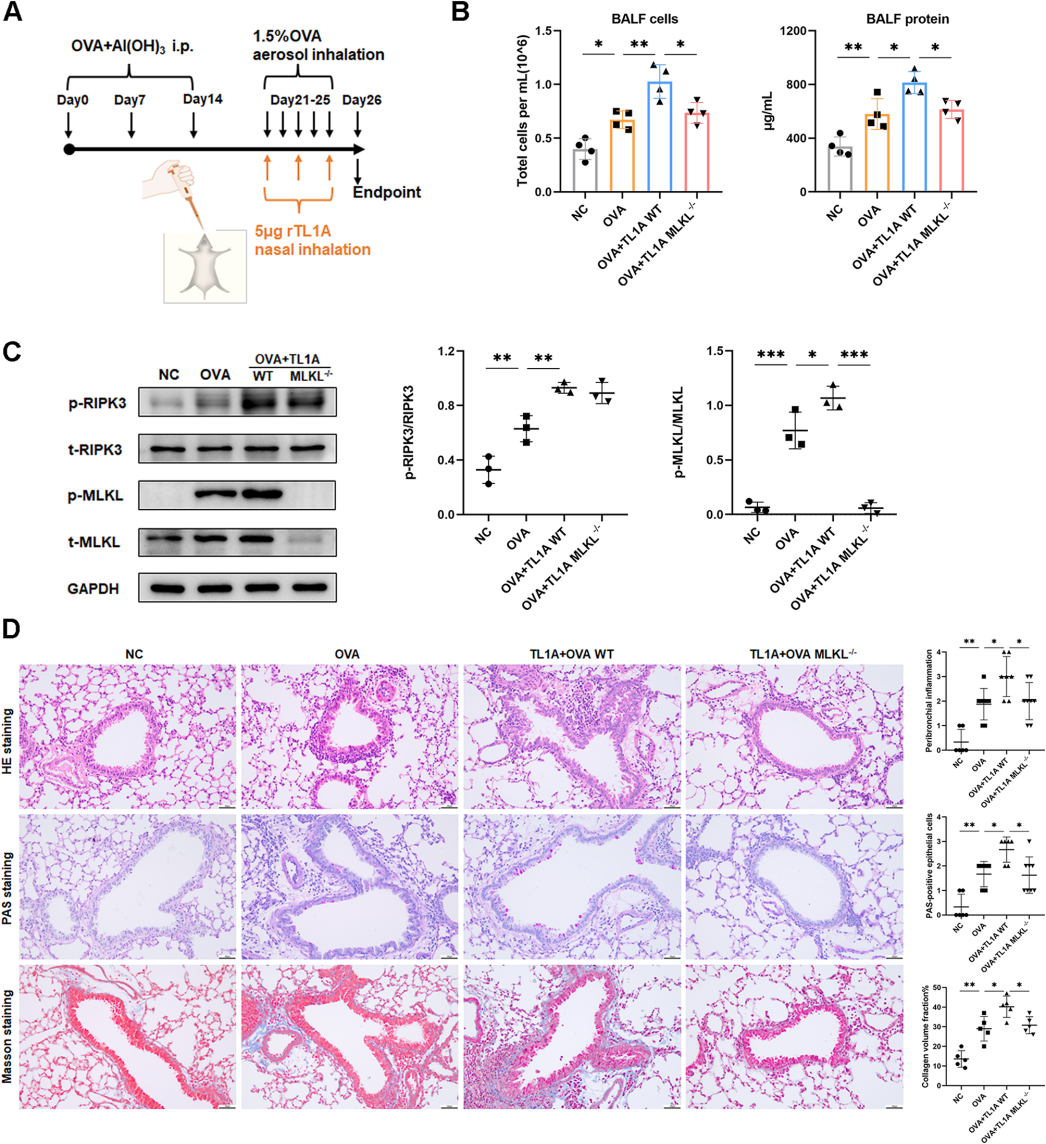
TL1A-induced necroptosis promotes asthma inflammation. **(A)** OVA-induced asthma mouse model experimental scheme and recombinant protein TL1A dosing schedule. **(B)** Total number of cells and total protein content in BALF (*n* = 4). **(C)** Western blot analysis of p-RIPK3, t-RIPK3, p-MLKL, and t-MLKL in lungs mice (*n* = 3). **(D)** Representative photomicrographs for lung paraffin sections from mice stained with H&E, PAS, and Masson staining, along with quantification of inflammation scores, PAS-positive cells, and collagen deposition around the airways (*n* = 5–8). Bars = 50 μm.*p < 0.05, **p < 0.01, ***p < 0.001

To explore the potential impact of TL1A on the regulation of OVA-induced necroptosis and airway inflammation, we administered TL1A intranasally to a mouse model of asthma (Fig. 5A). In WT mice, treatment with OVA and TL1A resulted in a significant increase in total cells and protein in the BALF compared to mice treated with OVA alone, which was attenuated by MLKL knockout (Fig. 5B). TL1A administration exacerbated OVA-induced lung necroptosis in mice, as determined by Western blot analysis (Fig. 5C). Further examination of lung sections using H&E, PAS, and Masson trichrome staining revealed a decrease in parabronchial inflammatory cell infiltration, mucus production, and collagen deposition in TL1A- and OVA-treated MLKL^-/-^ mice as compared to WT mice (Fig. 5D). The histological data support the conclusion that TL1A-induced necroptosis plays a vital role in asthma inflammation and airway remodeling.

### Necroptosis affects the airway epithelial barrier by disrupting intercellular adhesion

**Fig. 6:**
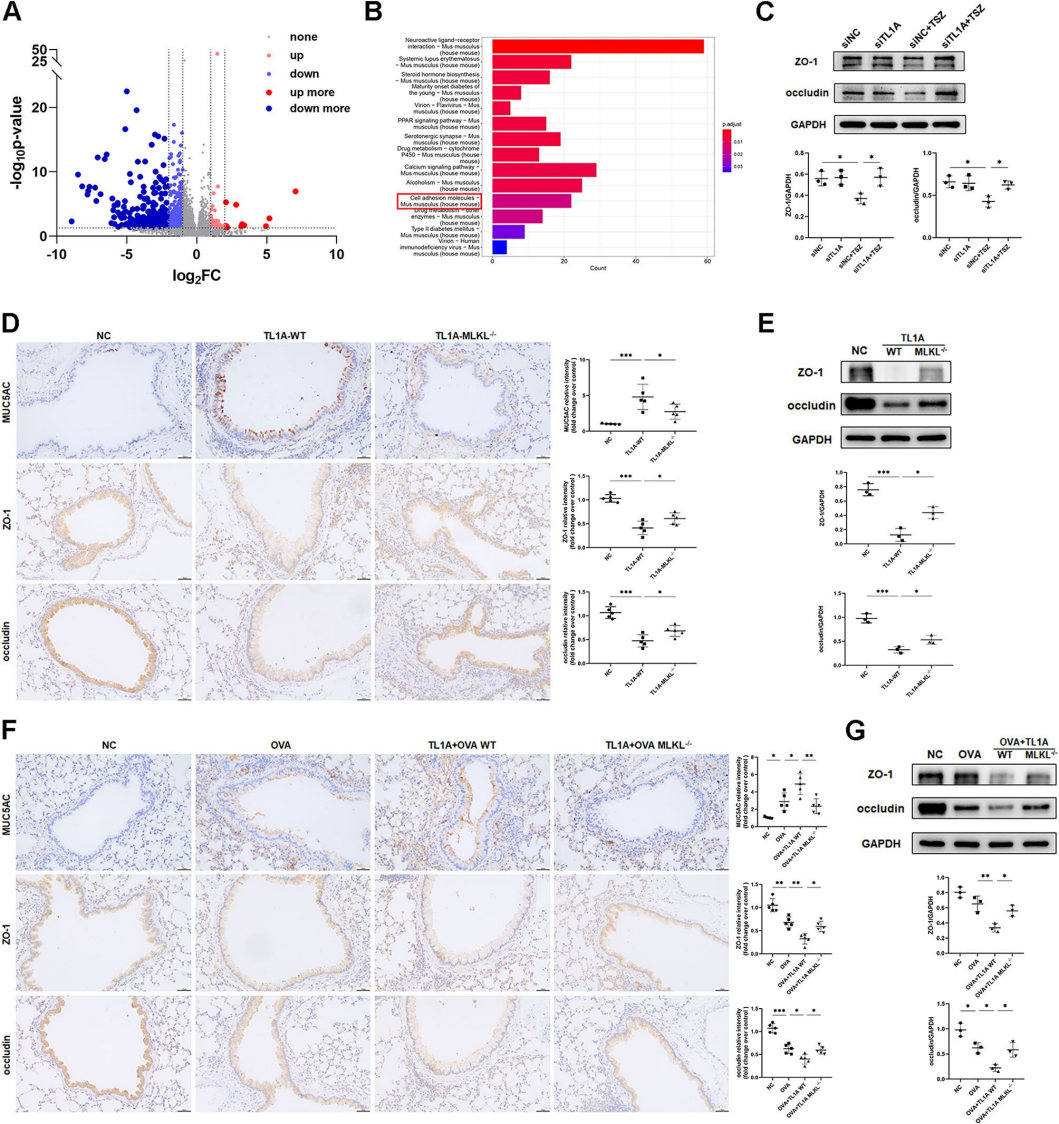
TL1A-induced necroptosis affects airway epithelial intercellular adhesion. **(A)** Volcano plot of RNA-seq from OVA-treated lung tissues between MLKL WT (*n* = 3) and knockout (*n* = 3) mice. (*P* < 0.05, log|FC|≥1) **(B)** Bubble map of KEGG pathway enrichment. **(C)** HBE cells were transfected with siTL1A and then stimulated by TSZ for 4 h. Occludin and zonulin-1 (ZO-1) expression were determined by Western blot (*n* = 3). **(D)** Representative images of MUC5AC, ZO-1, and occludin immunohistochemical staining in lungs of mice (*n* = 5). **(E)** Western blot analysis of occludin and ZO-1 in lungs of mice (*n* = 3). **(F)** Representative images of MUC5AC, ZO-1, and occludin immunohistochemical staining in lungs of mice (*n* = 5). **(G)** Western blot analysis of occludin and ZO-1 in lungs of mice (*n* = 3). Bars = 50 μm.*p < 0.05, **p < 0.01, ***p < 0.001

To further investigate the role of necroptosis signaling in asthma progression and its impact on downstream mechanisms, we performed mRNA profile analysis on WT and MLKL knockout OVA-induced mice, screening 505 down-regulated and 48 up-regulated genes (Fig. 6A). KEGG pathway enrichment analysis was performed on these genes revealing significant enrichment of cell adhesion molecule signaling pathway (Fig. 6B). We analyzed the expression of cell adhesion molecules occludin and ZO-1. Occludin and ZO-1 protein expression were diminished in HBE cells when exposed to TSZ, but TL1A knockdown helped to alleviate this effect (Fig. 6C). In vivo experiments showed that TL1A inhalation significantly reduced occludin and ZO-1 protein expression, which was restored after MLKL knockout (Fig. 6E). Immunohistochemical analysis showed similar changes in occludin, ZO-1, and MUC5AC in the airway epithelium (Fig. 6D), while the alveolar epithelium was not affected (Supplementary Fig. 3B). To characterize the impact of necroptosis on cellular junction molecules in asthma, we next analyzed the expression levels of occludin and ZO-1 in TL1A- and OVA-challenged mice. We found that necroptosis had a significant effect on the expression of these molecules. In the OVA-challenged group, levels of occludin and ZO-1 in airway epithelium were significantly reduced, which was further exacerbated by the presence of TL1A. Deletion of MLKL partially restored occludin and ZO-1 expression (Fig. 6F and G). However, these indicators were not affected in the alveolar epithelium (Supplementary Fig. 3C). Additionally, MLKL deletion also reduced the worsening of MUC5AC expression caused by TL1A (Fig. 6F). GSK872 blockade of necroptosis also had the same effect on occludin as MLKL deletion (Supplementary Fig. 3A).

#### TL1A-induced necroptosis impacts cell junction molecules via NF-κB activation

**Fig. 7:**
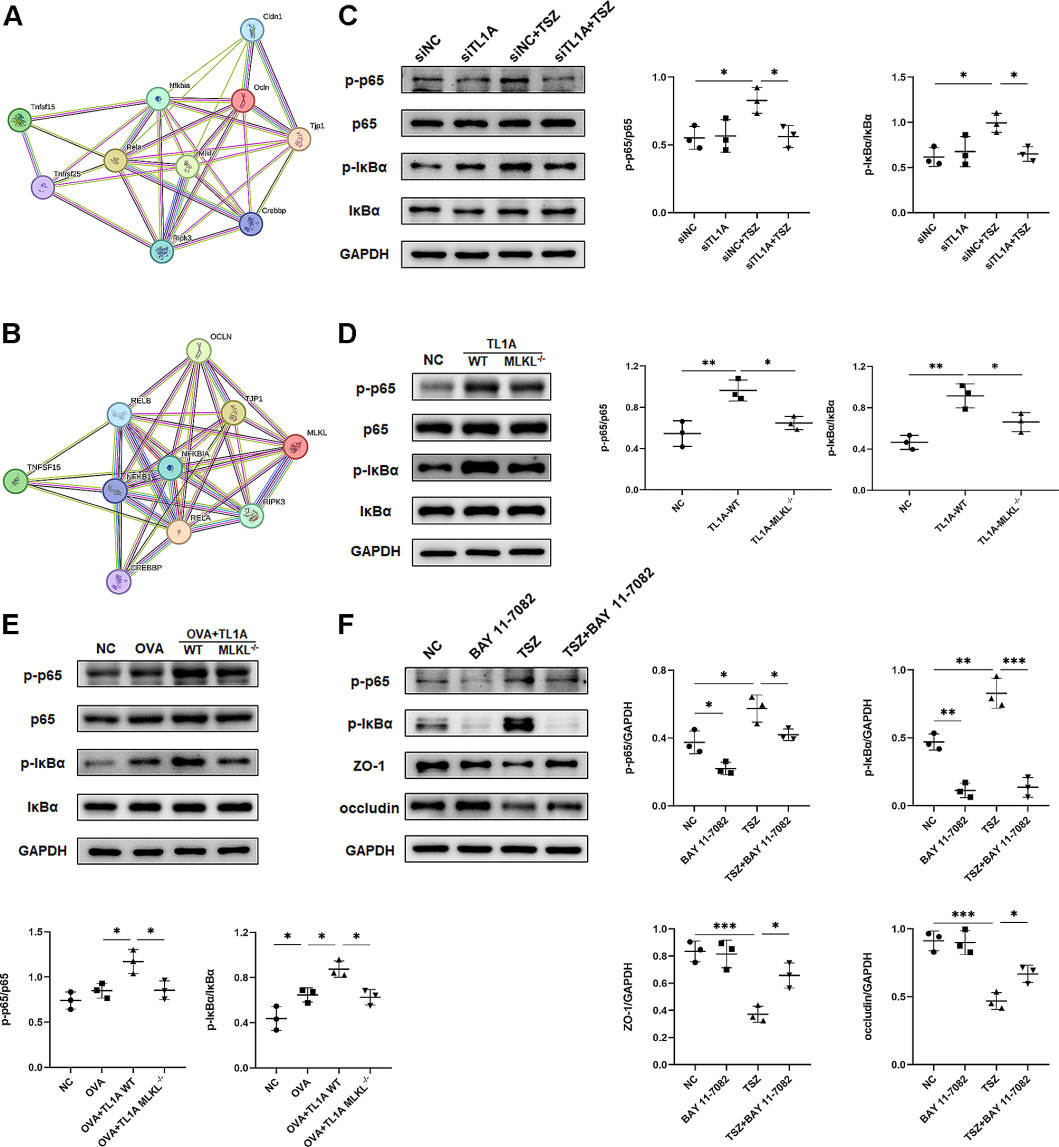
TL1A-induced necroptosis impacts cell junction molecules via NF-kB activation. **(A)** Human protein-protein interaction (PPI) network of necroptosis, NF-κB, and cell adhesion molecules. **(B)** Mouse PPI network of necroptosis, NF-κB, and cell adhesion molecules. **(C)** HBE cells were transfected with siTL1A and then stimulated by TSZ for 4 h. Phosphorylation of p65 and IκBα expression were determined by Western blot. **(D, E)** Western blot analysis of p-p65, p65, p-IκBα, and IκBα in lungs of mice. **(F)** HBE cells were treated with the IκBα inhibitor BAY 11-7082 for 1 h before TSZ for 4 h. The expression of p-p65, p-IκBα, ZO-1, and occludin was determined by Western blot. *N* = 3. *p < 0.05, **p < 0.01, ***p < 0.001

Previous studies have shown that the NF-κB signaling pathway is activated after the initiation of necroptosis. The STRING database was used to analyze the protein-protein interaction (PPI) network of TL1A, necroptosis, NF-κB, and cell adhesion molecules. In humans, there were 10 protein interactions with 37 connecting edges. The average local clustering coefficient for these interactions was 0.90 (Fig. 7A). In mice, there were 10 protein interactions, with 33 connecting edges that interacted with each other and had an average local clustering coefficient of 0.859 (Fig. 7B). Activation of necroptosis in HBE cells led to increased phosphorylation of p65 and IκBα in the NF-κB pathway. Silencing TL1A attenuates NF-κB activation in necroptosis-induced conditions (Fig. 7C). However, it is unclear whether this effect is due to blocking TL1A/DR3 signaling or blocking necroptosis activation. In vivo, TL1A induced phosphorylation of p65 and IκBα. After MLKL knockout, necroptosis was blocked, and TL1A-induced phosphorylation of p65 and IκBα was reduced (Fig. 7D). In addition, TL1A enhanced OVA-induced phosphorylation of p65 and IκBα, which was partially reverted by MLKL knockout (Fig. 7E). To determine whether cell junction proteins are affected by NF-κB pathway activation, we treated HBE cells with the IκBα inhibitor BAY 11-7082. The administration of BAY 11-7082 led to a noteworthy decrease in the expression of p-p65 and p-IκBα induced by TSZ, resulting in the reinstatement of occludin and ZO-1 (Fig. 7F).

## Discussion

In the absence or inhibition of caspase-8, cell death transitions from apoptosis to necrotic cell death, and the small-molecule inhibitor Nec-1 blocks this process. Based on this cornerstone, necroptosis was officially named as a non-apoptotic programmed cell death in 2005 [18, 19]. Over the past two decades, extensive academic researches have focused on the expansion of the cell death paradigm and the investigation of its precise mechanisms. Since necroptosis is a cytolytic death, it can lead to inflammation through the release of cellular contents and damage-associated molecular patterns (DAMPs), and the effects of MLKL on cells may contribute to the immunogenic effects of necroptosis [20]. We have demonstrated the upregulation and activation of necroptosis-dependent proteins, RIPK3 and MLKL, in the airway epithelia of asthma patients and animal models. Inhibition of RIPK3 or deletion of MLKL effectively attenuated parabronchial inflammation, mucus hypersecretion, and collagen fiber accumulation in the airways of asthmatic mice. RIPK3 blockade also inhibited the release of type 2 inflammatory factors. These findings are consistent with the effect of Nec-1 inhibition on RIPK1 treatment [21]. The presented data highlights the potential involvement of necroptosis in airway epithelial changes. Thus, identifying the exact mechanism involved may lead to new therapeutic strategies for the treatment of asthma.

TL1A is an attractive therapeutic target for the treatment of epithelial inflammation associated with chronic bowel disease and asthma [9, 11, 22]. At the same time, the existing literature highlights the potential of TL1A blockade to prevent or even reverse colon and lung fibrosis [23, 24]. Allergen-induced eosinophilic inflammation is associated with upregulation of the TL1A/DR3 axis and increased ILC2 activation in the airways of patients with mild asthma. In severe eosinophilic asthma, the TL1A/DR3 axis may be a co-stimulatory pathway that promotes airway eosinophil persistence through activation of ILC2s, particularly in patients susceptible to autoimmune responses [10]. Additionally, TL1A signaling through DR3 also enhances the pathogenicity of Th9 cells in allergic reactions [25]. Therefore, the involvement of TL1A/DR3 in eosinophilic inflammation and airway fibrosis associated with asthma indicates that it is a potential target for therapy.

The evidence for TL1A inducing necroptosis through its receptor DR3 is currently limited. Our study using siTL1A and OE-TL1A in HBE cells demonstrated that TL1A/DR3 can act as a necroptosis death trigger in the context of caspase inhibition. However, membrane-bound TL1A alone could not induce necroptosis but only exacerbated the induction of necroptosis by TSZ. More convincingly, the recombinant TL1A protein was able to induce necroptosis in mice with or without OVA challenge. The pathological changes in asthma induced by recombinant TL1A were partially reversed by MLKL knockout. These results suggest that changes in allergic inflammation and airway fibrosis in TL1A-induced asthma are largely inseparable from the effects of necroptosis.

The bronchial epithelium serves as the first-line barrier responsible for maintaining airway homeostasis in the lung. The apical junctional complex between adjacent epithelial cells consists of tight junctions (TJs) and adhesion junctions (AJs) [26]. Exposure of airway epithelial cells to the dust mite allergen in vitro or inhalation of OVA in mice resulted in the destruction of epithelial TJs. Patchy TJs destruction was observed in bronchial biopsies from asthmatics, and immunostaining showed low expression of ZO-1 and occludin [27]. Airway epithelial homeostasis is maintained by the stability of the epithelial barrier. Disruption of barrier integrity promotes the endo/exo translocation of inhaled particles into the subepithelial space where they encounter innate and adaptive immune cells, leading to airway inflammation and immune responses [28, 29]. Furthermore, activated TH2 cells and ILC2s, along with their secreted cytokines IL-4 and IL-13, cooperate to compromise the epithelial TJ barrier, leading to significant degradation of the bronchial epithelial barrier [30, 31].

Through mRNA sequencing, we predicted and subsequently validated the involvement of cell adhesion molecule signaling pathways in the detrimental effects of necroptosis on airways, both in vivo and in vitro. Previous research has shown that TL1A levels are increased in individuals with asthma. This study confirms that TL1A causes necroptosis and decreases the expression of tight junction proteins ZO-1 and occludin, thereby disrupting the function of the airway barrier. Currently, there is a dearth of research on the effect of necroptosis on the airway epithelial barrier. Similar to our data in asthma, a recent study indicates that in a piglet model of intestinal injury, pretreatment with Nec-1 inhibited LPS-induced necroptosis, improved the reduced expression of jejunal tight junction proteins claudin-1 and occludin protein, and ameliorated barrier function [32].

Furthermore, disruption of airway epithelial cell tight junctions by TL1A-induced necroptosis has been observed and activation of the NF-κB pathway may be involved in this process. The interaction between TL1A and DR3 has been reported to potentiate early NF-κB and late p38 MAP kinase phosphorylation, in recombinant systems or in cells naturally expressing DR3 [33–37]. Silencing TL1A expression can block NF-κB activation through TL1A/DR3 pathway. However, this effect plays a negligible role in the face of the powerful inflammatory response induced by cellular rupture and necrosis induced by TSZ. Interestingly, the release of endogenous DAMPs, such as ATP and HMGB1, induced by RIPK3/MLKL-mediated cell lysis, was not sufficient to prime CD8 + T cells. Instead, transcriptional activation of NF-κB in the dying cell was necessary to achieve T-cell priming [38]. NF-κΒ regulates the expression of many pro-inflammatory genes that may actively promote immunogenicity in dying cells [39]. Vucur et al. demonstrated that simultaneous activation of the necrosome and NF-kB in hepatocytes does not immediately lead to cell death. Instead, it forces the cells into a prolonged ‘sublethal’ state, characterized by leaky membranes and the release of specific chemokines [40]. Therefore, further investigation is necessary to explore the relationship between different forms of necroptosis and NF-κB.

One of the limitations of this study is that it is difficult to obtain BALF samples from patients with asthma, and serum levels of RIPK3 and p-MLKL may not be fully representative or conclusive. Additionally, we were unable to find human recombinant TL1A protein with stable activity that could induce necroptosis in HBE cells, which prevented us from conducting more detailed mechanistic studies in vitro. Furthermore, blocking necroptosis with GSK872 and knocking out MLKL both preceded OVA-challenge, indicating that blocking necroptosis may prevent airway damage in asthma. However, it is unclear whether it is effective in reducing established asthma changes. Further research is needed to clarify the clinical therapeutic role of necroptosis blockade after asthma onset. Next, we intend to investigate the mechanism of TL1A/DR3-induced necroptosis signaling in asthmatic airway epithelium using our epithelial cell-specific DR3 knockout mice.

Recently, the role of necroptosis in lung disease has received increasing attention as a growing number of researches indicate that programmed necrosis is associated with eosinophil- and neutrophil-associated inflammatory responses in the lung as well as lung epithelial cell injury. Eosinophils are more susceptible to necroptosis than neutrophils due to higher expression of RIPK1 and lower expression of caspase-8 [41]. The phenomenon of necroptosis selectively targeting eosinophils rather than neutrophils is associated with the pathological process of airway inflammation, epithelial damage, and remodeling [21]. It has been shown that neutrophils in the airways of neutrophilic asthmatic mice also undergo necroptosis and contribute to the formation of neutrophil extracellular traps that exacerbate airway inflammation in asthmatic mice [42]. In addition, recent studies have shown that the MLKL inhibitor GW806742X can block IL-33 secretion from bronchial epithelial cells both in vitro and in vivo, and can reduce eosinophilic airway inflammation in an allergic inflammation model [43]. Additionally, PTRF-IL-33-ZBP1 signaling has been found to mediate macrophage necroptosis and is involved in HDM-induced airway inflammation [44]. To date, an increasing number of studies have confirmed the important role of necroptosis in asthma. However, necroptosis has not been extensively studied and clinical evidence linking necroptosis to asthma is scarce. This limits the potential of necroptosis as a therapeutic target for asthma.

## Conclusion

In conclusion, this work reveals a novel role for TL1A in exacerbating airway inflammation and barrier disruption by inducing airway epithelial necroptosis. The study expands the current understanding of the TL1A/DR3-necroptosis axis in asthma pathogenesis and provides new perspectives into asthma therapeutic targets.

### Electronic supplementary material

Below is the link to the electronic supplementary material.

Supplementary Material 1

Supplementary Material 2

Supplementary Material 3

Supplementary Material 4

Supplementary Material 5

Supplementary Material 6

## Data Availability

No datasets were generated or analysed during the current study.

## Abbreviations

TL1A: Tumor necrosis factor-like cytokine 1 A
RIPK1: Receptor-interacting serine/threonine-protein kinase 1
RIPK3: Receptor-interacting serine/threonine-protein kinase 3
MLKL: Mixed lineage kinase domain-like protein
AEC: Airway epithelial cell
DR3: Death receptor 3
ILC2: The group 2 innate lymphoid cell
TNFR1: Tumor necrosis factor receptor-1
TLR3: Toll-like receptor 3
DAMPs: Damage-associated molecular patterns
MCP-1: Monocyte chemotactic protein-1
TJs: Tight junctions
AJs: Adhesion junctions
OVA: Ovalbumin
ZO-1: Zonula occludens-1
