# Supplementary figures and images for: Necroptosis plays a role in TL1A-induced airway inflammation and barrier damage in asthma
Source: Respir Res. 2024 Jul 10;25:271. doi: 10.1186/s12931-024-02900-4 (PMC11238433; doi:10.1186/s12931-024-02900-4)

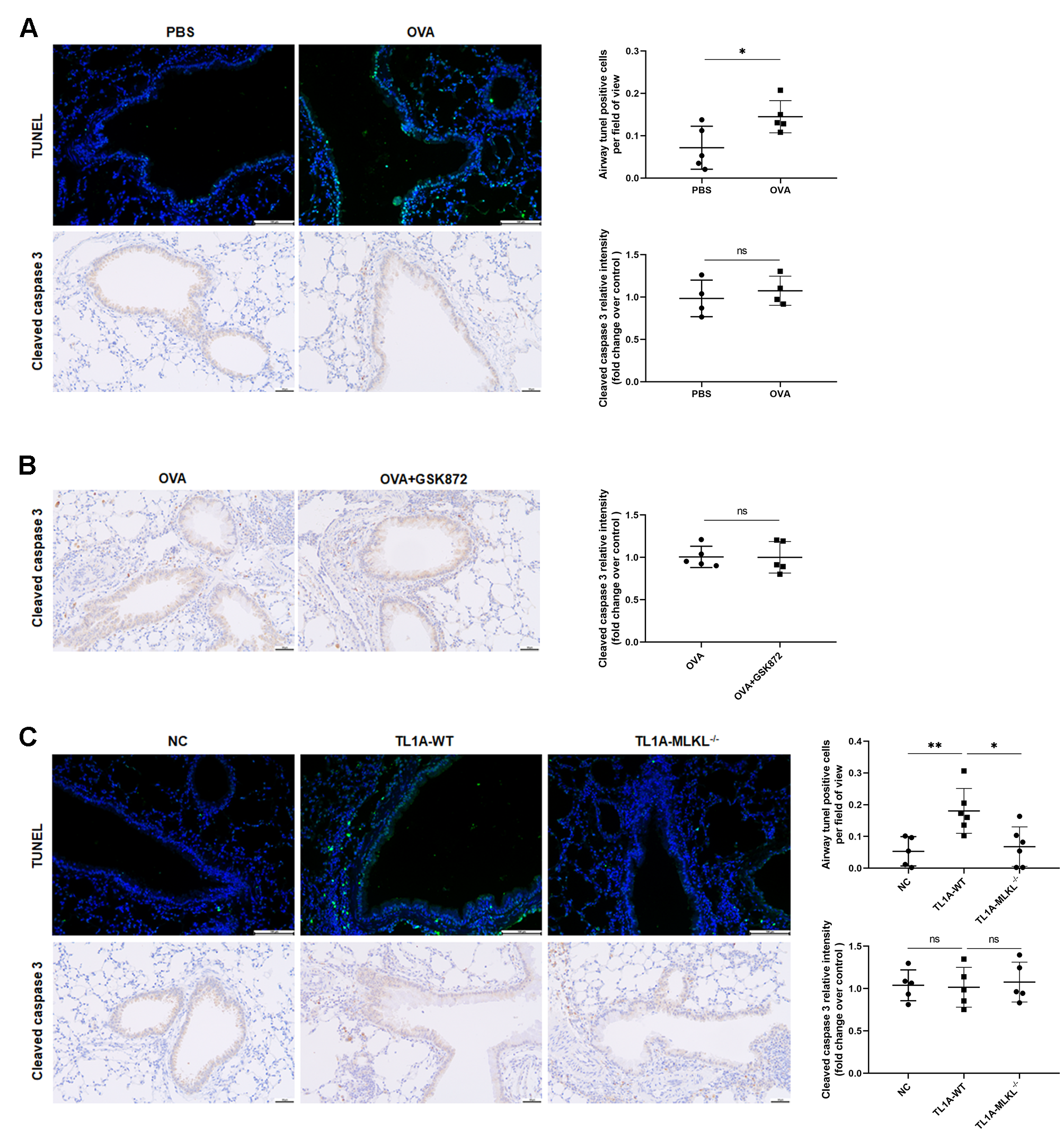

Supplement: Supplementary file 3 — Supplementary Material 3 [file 12931_2024_2900_MOESM3_ESM.tif]

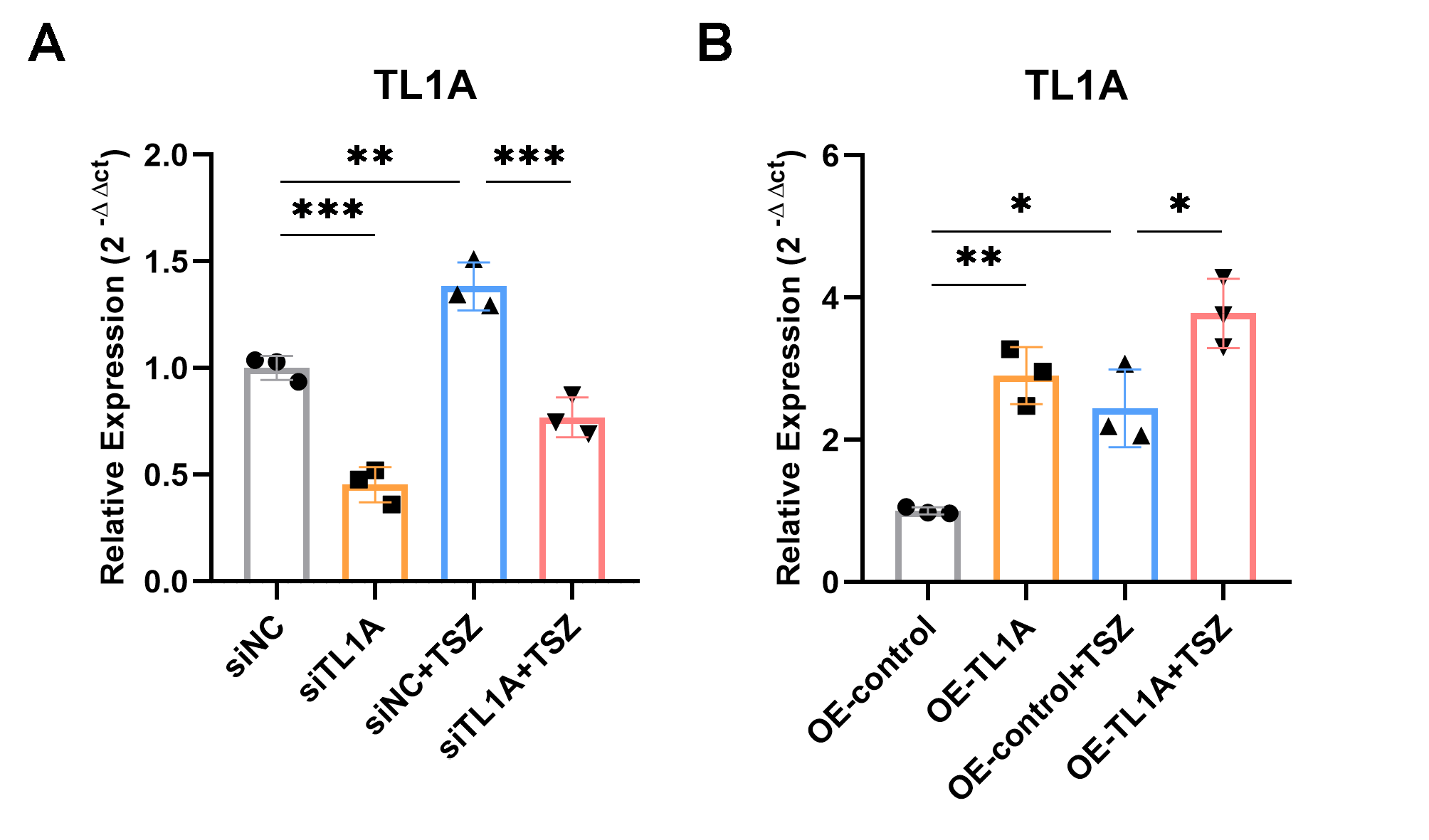

Supplement: Supplementary file 4 — Supplementary Material 4 [file 12931_2024_2900_MOESM4_ESM.tif]

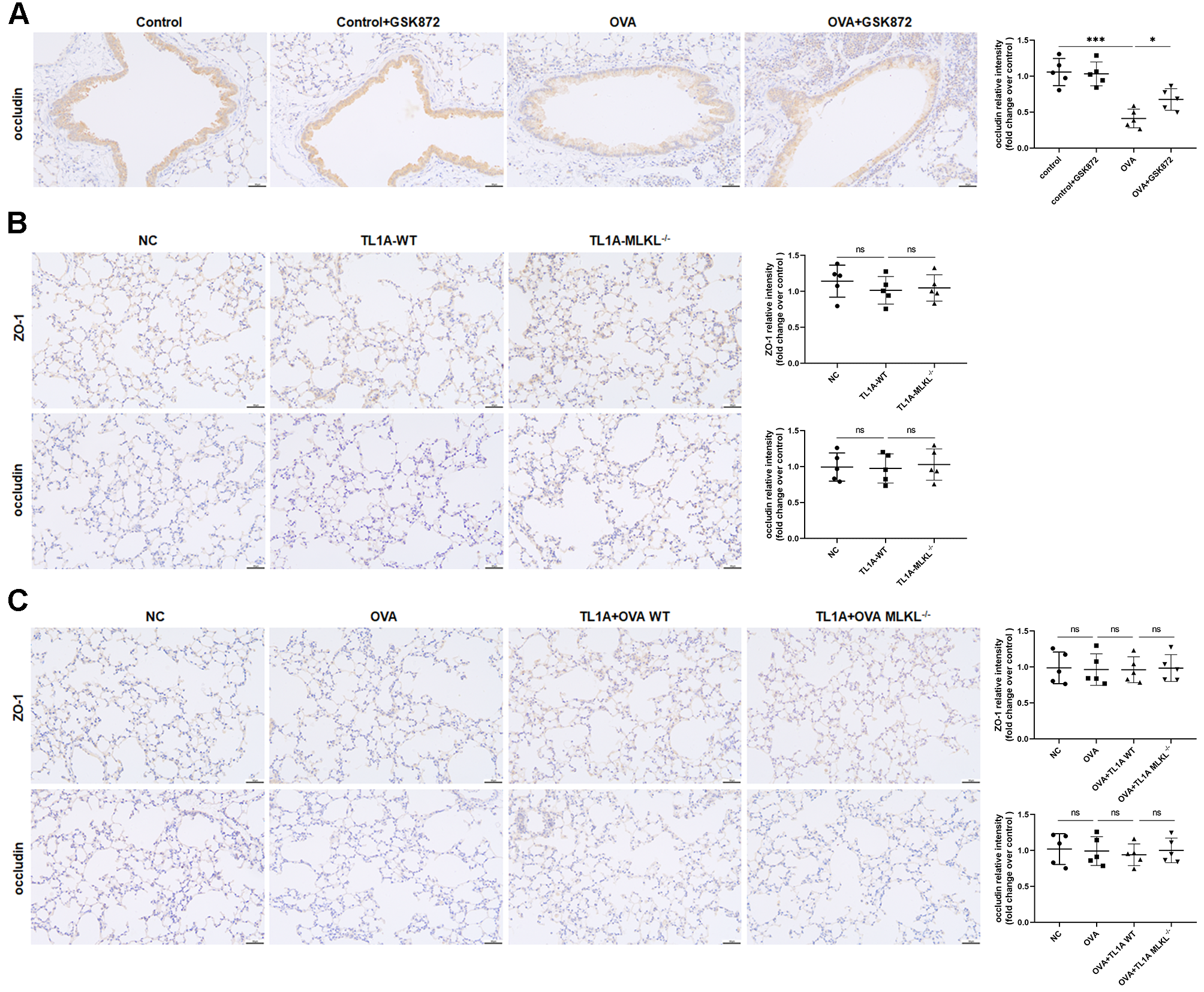

Supplement: Supplementary file 5 — Supplementary Material 5 [file 12931_2024_2900_MOESM5_ESM.tif]
