# Supplementary material for: Necroptosis plays a role in TL1A-induced airway inflammation and barrier damage in asthma
Source: Respir Res. 2024 Jul 10;25:271. doi: 10.1186/s12931-024-02900-4 (PMC11238433; doi:10.1186/s12931-024-02900-4)
Supplement: Supplementary file 6 — Supplementary Material 6 [file 12931_2024_2900_MOESM6_ESM.docx]

**Supplementary Information**

**Supplementary Table 1 Primers for RT-PCR**

**Supplementary Table 2 Characteristics of human serum subjects for ELISA**

**Supplementary Figure 1 OVA and TL1A treatments induce necroptosis but not apoptosis**

**(A)** Representative images of TUNEL staining and cleaved caspase 3 staining in lungs of mice (n=4-5). **(B)** Representative images of cleaved caspase 3 staining in lungs mice (n=5). **(C)** Representative images of TUNEL staining and cleaved caspase 3 staining in lungs of mice (n=5-6). Bars=50 μm.*p<0.05, **p<0.01, ***p<0.001, and ns. non-significant.

**Supplementary Figure 2 Efficiency of knockdown and overexpression of TL1A**

**(A**, **B)** The mRNA expression of TL1A was measured by RT-PCR relative to the expression of reference genes GAPDH. *p<0.05, **p<0.01, ***p<0.001.

**Supplementary Figure 3 OVA and TL1A treatment has no significant effect on alveolar epithelial occludin and ZO-1 expression**

**(A)** Representative images of occludin staining in lungs mice. **(B)** Representative images of ZO-1 and occludin immunohistochemical staining in lungs of mice. **(C)** Representative images of ZO-1 and occludin immunohistochemical staining in lungs of ^-^ mice. N=5. Bars=50 μm.*p<0.05, **p<0.01, ***p<0.001, and ns. non-significant.
